# Delayed Re-epithelialization Following Full-Field Er:YAG Laser Resurfacing: A Case Report

**DOI:** 10.7759/cureus.93281

**Published:** 2025-09-26

**Authors:** Hasan KH A H A Ashkanani, Abdulaziz AlRasheed, Wael AlDaraji

**Affiliations:** 1 Dermatology, Amiri Hospital, Kuwait City, KWT; 2 Plastic and Reconstructive Surgery, Kuwait Hospital, Kuwait City, KWT; 3 Dermatopathology, Dr Gera Tagani Research Centre, Newcastle University, Newcastle, GBR

**Keywords:** aesthetic medicine, allergy, er:yag laser, kuwait, laser skin resurfacing

## Abstract

Full-field erbium-doped yttrium aluminum garnet (Er:YAG) laser resurfacing is widely used for photoaging and scars, with re-epithelialization expected within five to seven days. Delayed healing is uncommon and may result from infection, inappropriate wound care, or allergic contact dermatitis (ACD). We present a 32-year-old woman who underwent full-face Er:YAG resurfacing (fluence: 8 J/cm², pulse duration: 300 µs, four passes, depth: 600 µm). Despite standard postoperative instructions, she substituted petrolatum ointment with a lanolin-containing moisturizer. At day seven, erosions and crusting persisted. Cultures and potassium hydroxide preparations were negative. Patch testing with the European Baseline Series and supplementary wound-care allergens (total: 45) demonstrated a strong positive reaction to lanolin (wool alcohols, 30% petrolatum), confirming ACD. Treatment included discontinuing the lanolin-containing product, substituting with plain petrolatum, initiating clobetasol propionate ointment, and prescribing a seven-day course of oral prednisone (30 mg daily). Rapid improvement followed, with complete epithelialization by day 21 and no scarring or pigment alteration. This case demonstrates lanolin-induced ACD as a cause of delayed epithelialization after laser resurfacing. Although negative microbiological studies cannot exclude infection, the strong positive patch test confirmed lanolin as the causative agent. Reporting of laser settings, diagnostic limitations, and wound care regimens is critical in case reports to enable reproducibility and contextual understanding. Patient education and careful postoperative product selection are vital to avoid preventable complications. Lanolin allergy should be considered in cases of delayed wound healing after ablative resurfacing. Early patch testing and substitution with inert, lanolin-free petrolatum can accelerate recovery. Clinicians must emphasize evidence-based wound care, avoidance of sensitizers, and prompt evaluation when healing deviates from the expected course.

## Introduction

Ablative laser resurfacing with carbon dioxide (CO₂) and erbium-doped yttrium aluminum garnet (Er:YAG) lasers is a cornerstone treatment for photoaging, facial rhytides, and acne scarring. These lasers achieve precise epidermal and dermal ablation through the principle of selective photothermolysis - a targeted photothermal reaction based on chromophore-specific absorption - which in turn stimulates neocollagenesis and promotes re-epithelialization. Er:YAG lasers, operating at a wavelength of 2,940 nm, are characterized by high water absorption and minimal thermal damage, enabling more superficial ablation than CO₂ lasers and facilitating faster recovery with fewer pigmentary complications [1,2]. Re-epithelialization following full-field Er:YAG laser treatment typically occurs within five to seven days in healthy individuals receiving proper postoperative care. Moist wound healing using petrolatum-based occlusive ointments is critical in supporting keratinocyte migration and restoring the skin barrier [2]. In contrast, non-occlusive environments may prolong inflammation and hinder epidermal regeneration [3,4].

This report presents a case of delayed re-epithelialization following full-field Er:YAG resurfacing, linked to inappropriate aftercare. The complication was corrected through targeted interventions, including elimination of a contact allergen and corticosteroid therapy. The patient provided informed consent for publication of her case.

## Case presentation

A 32-year-old female flight attendant from Tirana, Albania (Fitzpatrick skin type III) with no medical comorbidities underwent full-face Er:YAG laser resurfacing for photoaging. The procedure, performed with an Er:YAG platform in full-field mode, achieved approximately 600 microns of ablation to the deep papillary dermis. Immediate post-procedure response included uniform erythema and serous oozing.

The patient was instructed to apply a petrolatum-based occlusive ointment multiple times daily. Instead, she used a non-occlusive cosmetic cream and thermal spring water spray. No occlusive barrier was applied. By day seven, she presented with moderate crusting and incomplete wound healing. Examination revealed focal erosions and delayed epithelialization. Vital signs were stable and baseline laboratory studies were normal (Table 1).

**Table 1:** Patient's vital signs and laboratory test results (postoperative day seven).

Parameter	Result	Reference Range
Temperature	37.0 °C	36.5–37.5 °C
Blood pressure	118/75 mmHg	90/60–120/80 mmHg
Heart rate	80 bpm	60–100 bpm
Respiratory rate	16/min	12–18/min
WBC count	7.4 × 10⁹/L	4.0–11.0 × 10⁹/L
Hemoglobin	13.8 g/dL	12–16 g/dL (female)
Platelet count	252 × 10⁹/L	150–400 × 10⁹/L
C-reactive protein	2.1 mg/L	0.0–5.0 mg/L

Microbial cultures and potassium hydroxide (KOH) testing were negative (Table 2). These results were interpreted with caution, as negative fungal smears and cultures do not definitively rule out infection due to limited sensitivity, prior antimicrobial use, or technical factors. Similarly, bacterial cultures may yield false negatives depending on sampling and transport conditions. Patch testing excluded most allergens; however, the patient demonstrated a strong positive reaction to lanolin (wool alcohols) at both 48 and 96 hours, confirming allergic contact dermatitis (ACD). This finding directly implicated lanolin-containing ointments as a contributory factor to the delayed healing. Other allergens tested were negative.

**Table 2:** Results of microbiological investigations and patch testing. Abbreviations: KOH: potassium hydroxide; ACD: allergic contact dermatitis

Test	Result	Reference/Interpretation
Bacterial wound culture	Negative (no growth)	Negative does not exclude bacterial infection
Fungal culture	Negative (no growth)	Negative does not exclude fungal infection
KOH smear	Negative (no hyphae or spores visualized)	Negative does not exclude fungal infection
Patch testing (48 & 96 hrs)	Positive: lanolin (wool alcohols, 30% petrolatum)	Positive indicates allergic contact dermatitis (ACD)
Other patch test allergens (n=44)	Negative	No sensitization detected

This case required a multifaceted therapeutic adjustment to restore the expected healing trajectory, which started on day eight. On day eight, the treatment plan was revised. The patient was transitioned to plain white petrolatum ointment without additives or sensitizers, avoiding potential lanolin exposure. She was prescribed clobetasol propionate 0.05% ointment twice daily and oral prednisone 30 mg daily for seven days to attenuate inflammation and promote keratinocyte recovery. This approach was based on evidence that topical corticosteroids may reduce post-laser erythema and accelerate resolution without impairing epithelialization.

Substitution of the cosmetic moisturizer and thermal water with plain white petrolatum was prompted by the concern for lanolin-induced ACD. Lanolin, although widely used, has documented allergenic potential, particularly in patients with impaired skin barriers such as post-laser wounds. Several reports have identified certain cosmetic moisturizers as a sensitizing agent in postoperative wound healing, with delayed epithelialization attributed to lanolin content [5]. Clinical findings and response to therapy supported an inflammatory etiology, but histopathologic confirmation was not pursued.

**Figure 1:**
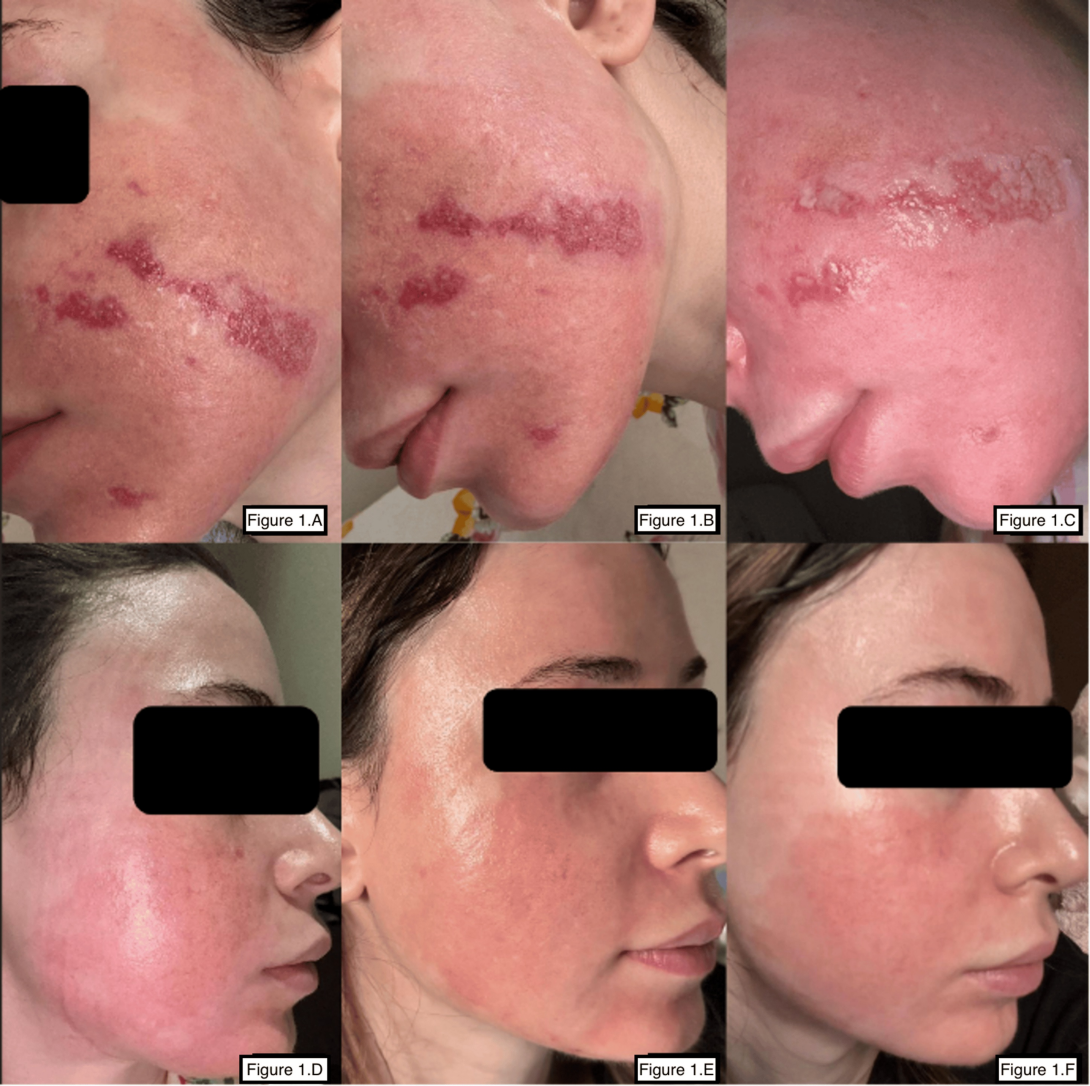
Sequential clinical photographs illustrating delayed and then resolved re-epithelialization following full-field Er:YAG resurfacing. Standardized digital photographs taken under consistent lighting and angle demonstrate the healing progression after full-field Er:YAG ablation. (1A) Day 7: Persistent crusting and erosions with incomplete epithelial coverage, indicating delayed wound healing. (1B) Day 9: Partial crust resolution with early evidence of epithelial regeneration following initiation of corticosteroid therapy. (1C) Day 12: Marked reduction in erosions and crusting, with visible new epidermis and residual inflammatory erythema. (1D) Day 14: Ongoing re-epithelialization; borders of erosions have softened and erythema appears more diffuse. (1E) Day 19: Near-complete epithelial coverage; fading erythema with no signs of secondary infection or pigment change. (1F) Day 21: Complete epithelialization; only mild background erythema persists without scarring or dyspigmentation. Interpretation: This figure demonstrates the temporal relationship between appropriate wound care, anti-inflammatory therapy, and recovery of the epidermal barrier.

Serial photographs documented the progression of healing. Initial post-laser erythema was followed by crust formation, erosions, and sharply demarcated inflammatory plaques corresponding to areas treated with non-occlusive moisturizers (Figure 1A). Following corticosteroid therapy and substitution of petrolatum ointment, rapid re-epithelialization occurred with near-complete resolution of erythema and no scarring by day 21 (Figures 1B-1F).

## Discussion

This case highlights how deviations from standard postoperative care can delay healing after Er:YAG resurfacing. The patient’s use of non-occlusive cosmetics disrupted moist wound healing and introduced a proven allergenic component. Patch testing confirmed a strong positive lanolin allergy, directly linking the lanolin-containing ointment to the delayed re-epithelialization. This strengthens the causal inference, as the patient improved rapidly after switching to lanolin-free petrolatum.

Negative cultures and KOH tests were acknowledged as limited in sensitivity, but the positive allergen test provided objective evidence supporting allergic contact dermatitis as the primary mechanism.

Laser parameters strongly influence outcomes. Excessive fluence, stacked pulses, or too many passes are known risk factors for delayed healing and scarring. Reporting of settings (fluence, pulse duration, number of passes, depth achieved) is essential to allow reproducibility and contextualization. In this case, parameters were within standard ranges, supporting the role of aftercare rather than overtreatment in the delayed epithelialization.

Postoperative wound care is equally critical. Standard evidence-based regimens include frequent gentle compresses with dilute acetic acid (0.25%), saline, or cooled boiled water several times daily, followed by application of bland occlusive ointment such as plain white petrolatum. Topical antibiotics are generally avoided due to high sensitization rates. Gentle cleansing with mild, fragrance-free cleansers is introduced after re-epithelialization is largely complete. Patient education is crucial, as inappropriate substitutions can compromise healing.

Topical and systemic corticosteroids are not routinely indicated post-laser, but when carefully timed and monitored, they play an important role in correcting excessive inflammation. Clobetasol diproprionate has demonstrated efficacy in reducing post-procedural erythema and minimizing pigmentary changes without hindering epithelialization [5,6]. However, the use of high-potency topical and systemic corticosteroids in the immediate post-laser period remains controversial, as excessive immunosuppression may risk impaired re-epithelialization, hypopigmentation, or secondary infection, necessitating cautious patient selection and close monitoring.

Additionally, increased awareness of contact allergens in postoperative care is vital. Lanolin was named the American Contact Dermatitis Society’s "Allergen of the Year" in 2023, reflecting its relevance in patients with compromised barriers [7]. Plain petrolatum remains the gold standard for occlusive therapy due to its inert profile and absence of sensitizers.

## Conclusions

This case highlights the importance of adherence to evidence-based wound care following laser resurfacing procedures. Delayed healing was effectively managed by eliminating lanolin-containing products and initiating anti-inflammatory therapy. Educating patients on proper post-procedural care and recognizing when to escalate treatment are essential in optimizing outcomes and avoiding complications.
